# Zinc supplementation affects favorably the frequency of migraine attacks: a double-blind randomized placebo-controlled clinical trial

**DOI:** 10.1186/s12937-020-00618-9

**Published:** 2020-09-14

**Authors:** Hedieh Ahmadi, Seyedeh Shabnam Mazloumi-Kiapey, Omid Sadeghi, Morteza Nasiri, Fariborz Khorvash, Tayebeh Mottaghi, Gholamreza Askari

**Affiliations:** 1 Department of Community Nutrition, School of Nutrition and Food Science, Isfahan University of Medical Sciences, P.O. Box 8174673461, Isfahan, Iran; 2 Department of Nutrition, Faculty of Medicine, Mashhad University of Medical Sciences, Mashhad, Iran; 3 Students’ Scientific Research Center, Tehran University of Medical Sciences, Tehran, Iran; 4 Department of Community Nutrition, School of Nutritional Sciences and Dietetics, Tehran University of Medical Sciences, Tehran, Iran; 5 Student Research Committee, School of Nursing and Midwifery, Shiraz University of Medical Sciences, Shiraz, Iran; 6 Department of Operating Room Nursing, School of Nursing and Midwifery, Shiraz University of Medical Sciences, Shiraz, Iran; 7 Neurology Research Center, School of Medicine, Isfahan University of Medical Sciences, Isfahan, Iran

**Keywords:** Zinc, Migraine, Headache, Clinical trial

## Abstract

**Background:**

Observational studies have shown a link between zinc deficiency and migraine headaches. We aimed to examine the effect of zinc supplementation on the characteristics of migraine attacks in patients with migraine.

**Methods:**

This randomized clinical trial was conducted on 80 patients with migraine. Patients were *randomly* assigned to *receive either* zinc sulfate (220 mg/d zinc sulfate) or *placebo (*lactose*) for 8 weeks*. Anthropometric measures, serum zinc concentrations, and characteristics of migraine attacks (headache severity, frequency and duration of migraine attacks, and headache daily results) were assessed at baseline and end of the trial.

**Results:**

Compared with the placebo, zinc supplementation resulted in a significant reduction in headache severity (− 1.75 ± 1.79 vs. -0.80 ± 1.57; *P* = 0.01) and migraine attacks frequency (− 2.55 ± 4.32 vs. -0.42 ± 4.24; *P* = 0.02) in migraine patients. However, the observed reduction for headache severity became statistically non-significant when the analysis was adjusted for potential confounders and baseline values of headache severity. Other characteristics of migraine attacks including the duration of attacks and headache daily results were not altered following zinc supplementation either before or after controlling for covariates.

**Conclusion:**

Zinc supplementation had a beneficial effect on the frequency of migraine attacks in migraine patients. Additional well-designed clinical trials with a long period of intervention and different dosages of zinc are required.

**Trial registration code:**

IRCT20121216011763N23 at www.irct.ir.

## Introduction

Migraine is a chronic neurovascular disorder that affects 10–20% of general population around the world [1, 2]. Patients with this disorder suffer from severe headaches and also nausea, vomiting, photophobia, and phonophobia during a migraine attack [3, 4]. Migraine-related headaches are typically one-sided and throbbing that may last 4–72 h [5]. Migraine can adversely affect the patients’ quality of life and impose a high burden on the health care system [6].

Analgesic drugs are commonly used for alleviating the severity of migraine headaches. However, these drugs have several side effects [7]. Hence, several supplementary treatments have been suggested for the management of migraine symptoms [8–11]. Among these methods, supplementation with some micronutrients receives a great deal of attention [2, 9, 12, 13]. Based on the previous studies, taking vitamin B2, pyridoxine, folate, and magnesium can improve migraine symptoms [2, 9, 12, 13]. However, the effect of other micronutrients on these symptoms has not been well-known.

Zinc is an essential trace element with antioxidant [14–16] and anti-inflammatory [17, 18] properties. It plays a critical role in neuronal signaling and is also known as a cofactor for antioxidant enzymes [19]. Some observational studies have revealed a moderate rate of zinc deficiency among patients with migraine [20, 21]. Severe zinc deficiency can lead to neurological disorders including attention deficit disorder, lethargy, memory impairment, and learning difficulties [22]. Also, it has been shown that low serum concentrations of zinc are positively associated with migraine attacks [20]. Given the inflammatory nature of migraine and contributing **the** oxidative stress in this disorder, zinc may have a favorable effect on migraine attacks. However, to the best of our knowledge, no study has examined the effect of zinc supplementation on migraine symptoms or characteristics of migraine attacks. Hence, the current study was done to examine the effect of zinc supplementation on characteristics of migraine attacks in migraine patients.

## Material and methods

### Participant

This double-blind randomized clinical trial was conducted on migraine patients from January to April 2016. Migraine was diagnosed by an experienced neurologist according to the below-mentioned International Headache Society (IHS) criteria [23]:

1. At least five attacks fulfilling criteria (2) to (4).
2. Headache attacks lasting 4–72 h (untreated or unsuccessfully treated).
3. Headache has at least two of these characteristics: (a) unilateral location, (b) pulsating quality, (c) moderate or severe pain intensity, (d) aggravation by or causing avoidance of routine physical activity (e.g. walking or climbing stairs).
4. The presence of nausea, vomiting, photophobia, or phonophobia (at least one of the symptoms) during headache.
5. Not better accounted for by another ICHD-3 diagnosis.

In the current trial, we included migraine patients, with an age range between 20 and 60 years, those who were receiving the routine treatment for the management of migraine symptoms, and those who were willing to participate in the study. Also, we selected the patients who had > 5 years’ history of migraine or its symptoms. Patients with tension-type headaches and those with chronic kidney disease or metabolic disorders (diabetes and cardiovascular diseases) were excluded. Moreover, patients who were taking vitamin and mineral supplements (particularly zinc supplement), drugs for other diseases except for migraine disease, and oral contraceptive drugs during the last month were excluded. Patients who were current smokers or in a pregnancy or lactation period were excluded as well.

### Sample size calculation

On the basis of the sample size formula suggested for randomized clinical trials and by considering type I error of 5% (α = 0.05), type II error of 20% (β = 0.20, power = 80%), and serum concentrations of zinc as key variable [20], we determined a sample size of 33 individuals for each group. To get a more confident result and consider a dropout rate of 20%, we recruited 40 patients for each group.

### Ethical consideration

All participants fulfilled a written consent form before inclusion in the clinical trial. This study was approved by the Institutional Review Board and Ethics Committee of Isfahan University of Medical Sciences, Isfahan, Iran. In addition, we did the trial based on the guidelines laid down in the Declaration of Helsinki. This study was registered at the Iranian Registry of Clinical Trials website (IRCT, www.irct.ir) with the registration code of IRCT20121216011763N23.

### Intervention

Firstly, patients were stratified based on age (20–40 and 40–60 years), gender (male and female), and body mass index (18.5–24.9 and 25–30) into different blocks. Then, they were randomly allocated to the intervention or control groups using a lottery, by a person who was unaware of the aim of the study. Patients in the intervention group (*n* = 40) received the routine treatments for migraine patients in addition to the zinc sulfate capsule (produced by Alhavi Company) containing 220 mg zinc sulfate or 50 mg elemental zinc. Participants in the control group (*n* = 40) received the routine treatments in addition to the placebo capsule (containing lactose) that was produced in the School of Pharmacy, Isfahan, Iran. The packages of zinc and placebo capsules were similar in the terms of color, appearance, and taste and were given to the patients at the study baseline. The routine treatment [200/500 mg sodium valproate (such as Depakin), 50/100 mg sumatriptan, or 1 mg ergotamine] in the intervention group was similar to that administered in the control group. The duration of intervention was 8 weeks and patients in both groups were asked to give back the empty packages at the end of the trial. Outcome variables were assessed at the study baseline and end of the trial. Data on baseline variables, dietary intakes throughout the trial (dietary recalls), and outcome variables were collected by a second researcher who was unaware of the groups’ assignment. Participants’ compliance was calculated using this formula: (number of used packages/all given packages) × 100. Participants were followed weekly via a mobile phone message to ensure their adherence to the interventions. All participants were asked not to change their dietary intake, physical activity, and medications during the study. Moreover, participants were asked to avoid the intake of nicotine and headache-trigger foods such as caffeine-rich foods throughout the intervention. Also, they were asked to report any adverse event due to the consumption of zinc or placebo capsules.

To follow dietary intakes throughout the trial, we collected the three one-day dietary recalls (including two working days and a non-working day) at weeks 1, 6, and 8 from each participant by telephone interview [24]. To complete the dietary recalls, participants were asked to report dietary intakes based on household measures. Then, we converted household measures to grams using available booklets. We considered the average dietary intakes in these 3 days as participants’ dietary intakes during the intervention. To obtain nutrients intakes on the basis of the food recalls, we used the Nutritionist IV software (based on the US National Nutrient Databank) modified for Iranian foods. The reliability and validity of the dietary recall were confirmed previously [24, 25].

### Assessment of migraine attacks

The characteristics of migraine attacks including headache severity, frequency and duration of attacks, and headache daily results (HDR) were determined at baseline and end of the trial. To measure headache severity, the visual analog scale (VAS) was used. Based on this scale, the severity of headache ranks between 1 and 10 [26, 27]. The number of migraine attacks in a month was considered as the frequency of migraine attacks. Attacks duration was considered as the number of hours in which a migraine attack lasts. To determine the mean duration of migraine attacks per day, named HDR, the following formula was used: migraine attacks frequency × attack duration [27].

### Assessment of serum zinc levels

After a-12 h fasting, five cc blood sample was taken from each patient at baseline and end of the trial. Serum zinc concentrations were measured using the atomic absorption spectrophotometry (AAS) method. Based on this method, serum zinc levels of 70–125 μg/dL were considered as a normal level.

### Assessment of other variables

Required information on age, gender, education, having a history of other diseases, medication, body mass index (BMI), and supplement use was collected using a researcher-made questionnaire. BMI was calculated as weight in kilograms divided by height in square meters (kg/m^2^). Height was measured in a standing position without shoes using a tape measure with the nearest 0.5 cm. Weight was determined with minimal clothing and without shoes by an analog scale with a precision of 100 g.

### Statistical analysis

Kolmogrov-Smirnov test was used to examine the normal distribution of variables. To examine differences in qualitative and quantitative variables between groups, we used the Chi-square test and independent-sample t-test, respectively. In addition, the independent sample t-test was used to compare between-group changes for the main outcomes. To perform a within-group comparison, the paired-sample t-test was applied. Multivariate analysis of covariance (ANCOVA) was used to examine the effect of zinc supplementation on BMI, serum zinc concentrations, and characteristics of migraine attacks. In this analysis, baseline values of outcome variables as well as age, gender, baseline BMI, and dietary intake of zinc throughout the trial were adjusted to exclude the potential risk of bias and detect an independent effect. Dietary intake of zinc was adjusted because patients in the placebo group consumed zinc-containing foods greater than those patients in the zinc group. All statistical analyses were done using SPSS software version 18 (SPSS, Inc. Chicago, IL, USA). *P* < 0.05 was considered as significant level.

## Results

All patients in the zinc (*n* = 40) and placebo (*n* = 40) groups completed the trial and there was no dropout. In addition, the compliance rate in our study was high, such that 100% of capsules were taken throughout the trial in both groups.

Demographic variables and baseline characteristics of patients are shown in Table 1. No significant difference was found between the zinc and placebo groups in terms of age, gender, and education as well as baseline weight, BMI, serum zinc concentrations, and characteristics of migraine attacks.

**Table 1:** Baseline characteristics of migraine patients who received either zinc supplement or placebo

Variables	Zinc group^a^	Placebo group^b^	t	df	*P*-value^*^
Age (year)	37.40 ± 12.26	38.87 ± 13.69	−0.50	78.0	0.61
Gender (female) (%)	82.5	70.0	1.72	1	0.18
Education (university graduated) (%)	32.5	25.0	0.54	1	0.45
Weight (kg)	62.58 ± 12.48	66.00 ± 15.14	−1.10	78	0.27
BMI (kg/m^2^)	24.36 ± 4.19	25.50 ± 4.91	− 1.11	78	0.26
Zinc (μg/dL)	85.41 ± 12.87	84.20 ± 11.65	0.44	78	0.66
Characteristics of migraine attacks					
Headache severity^c^	7.22 ± 1.14	6.80 ± 0.93	1.81	78	0.07
Attacks frequency (per month)	8.80 ± 6.16	9.45 ± 5.39	−0.50	78	0.61
Attacks duration (hour)	17.67 ± 13.59	22.85 ± 16.47	−1.53	78	0.13
HDR^d^	151.67 ± 138.87	198.00 ± 138.98	−1.49	78	0.14

*Abbreviation*: *BMI* body mass index, *HDR* headache diary result, *VAS* visual analog scale

All values are presented as mean (SD) or percent

^a^Received zinc capsule containing 220 mg zinc sulfate one time per day

^b^Received placebo capsule containing lactose one time per day

^c^Measured by VAS that ranked headache severity from 1 to 10

^d^Determined by formula: frequency × duration

^*^Obtained from independent sample t-test or Chi-square test, as appropriate

Dietary intakes of participants throughout the trial in the zinc and placebo groups are presented in Table 2. There was no significant difference in dietary intake of energy, proteins, fats, calcium, magnesium, phosphorus, and fiber between the zinc and placebo groups. However, patients in the placebo group had higher intakes of dietary zinc and carbohydrate than those in the zinc group. Due to the importance of zinc intake throughout the trial, it was adjusted in the final analysis.

**Table 2:** Dietary intake of migraine patients who received either zinc supplement or placebo throughout the trial

	Zinc group^a^	Placebo group^b^	t	df	*P*-value^*^
Energy (Kcal)	1737.0 ± 183.7	1957.1 ± 148.4	−0.93	67	0.35
Proteins (g/d)	68.9 ± 6.7	75.6 ± 5.4	− 0.77	67	0.44
Carbohydrates (g/d)	78.6 ± 10.6	246.8 ± 22.3	− 6.78	48.6	< 0.001
Fats (g/d)	213.9 ± 30.2	154.6 ± 30.6	1.37	67	0.17
Calcium (mg/d)	934.0 ± 130.5	921.8 ± 149.1	0.06	67	0.95
Magnesium (mg/d)	293.5 ± 31.8	299.9 ± 27.1	−0.15	67	0.87
Phosphorous (mg/d)	1350.4 ± 124.4	1736.4 ± 246.0	−1.38	67	0.17
Zinc (mg/d)	8.1 ± 0.6	10.2 ± 0.7	− 2.27	67	0.02
Fiber (g/d)	16.0 ± 1.7	20.7 ± 2.9	−1.37	67	0.17

All values are presented as mean (SE)

^a^Received zinc capsule containing 220 mg zinc sulfate one time per day

^b^Received placebo capsule containing lactose one time per day

^*^Obtained from independent sample t-test

The effect of zinc supplementation on BMI, serum zinc concentrations, and characteristics of migraine attacks is indicated in Table 3. Compared with the study baseline, zinc supplementation resulted in a significant reduction in headache severity (5.47 ± 1.78 vs. 7.22 ± 1.14; *P* < 0.001), migraine attacks frequency (6.25 ± 4.38 vs. 8.80 ± 6.16′ *P* = 0.001), attacks duration (13.92 ± 9.90 vs. 17.67 ± 13.59; *P* = 0.005), and HDR (94.15 ± 117.42 vs. 151.67 ± 138.87; *P* < 0.001). Also, a significant increase in serum zinc concentrations was observed following zinc supplementation (89.52 ± 12.22 vs. 85.41 ± 12.87; *P* < 0.001). However, zinc supplementation had no significant effect on BMI. When comparing changes in the zinc group with the placebo group, we found a significant lowering effect of zinc supplementation on headache severity (− 1.75 ± 1.79 vs. -0.80 ± 1.57; *P* = 0.01) and migraine attacks frequency (− 2.55 ± 4.32 vs. -0.42 ± 4.24; *P* = 0.02), while we did not find any significant effect on serum zinc concentrations, attacks duration, and HDR. When the analyses were adjusted for BMI, age, gender, dietary intake of zinc during the trial, and baseline value of outcome variables, only the effect of zinc supplementation on migraine attacks frequency remained significant (Table 4).

**Table 3:** Serum zinc concentrations, BMI and characteristics of migraine attacks at baseline and 8 weeks after the intervention in migraine patients who received either zinc supplement or placebo

	Zinc group^a^			Placebo group^b^			t	df	*P*-value^*^
	Baseline	Week 8	Change	Baseline	Week 8	Change			
BMI (kg/m^2^)	24.36 ± 4.19	24.21 ± 4.07	−0.14 ± 0.72	25.50 ± 4.91	25.25 ± 5.33	−0.25 ± 1.65	0.36	78	0.71
Zinc (μg/dL)	85.41 ± 12.87	89.52 ± 12.22	4.11 ± 5.46^c^	84.20 ± 11.65	87.07 ± 12.26	2.87 ± 7.77^a^	0.82	78	0.41
Headache severity^d^	7.22 ± 1.14	5.47 ± 1.78	−1.75 ± 1.79^c^	6.80 ± 0.93	6.00 ± 1.50	−0.80 ± 1.57^a^	− 2.51	78	0.01
Attacks frequency (month)	8.80 ± 6.16	6.25 ± 4.38	− 2.55 ± 4.32^c^	9.45 ± 5.39	9.02 ± 6.37	− 0.42 ± 4.24	− 2.22	78	0.02
Attacks duration (hour)	17.67 ± 13.59	13.92 ± 9.90	−3.75 ± 7.91^c^	22.85 ± 16.47	18.65 ± 14.47	− 4.20 ± 12.04^a^	0.19	78	0.84
HDR^e^	151.67 ± 138.87	94.15 ± 117.42	− 57.52 ± 91.64^c^	198.00 ± 138.98	165.45 ± 155.59	− 32.55 ± 129.22	− 0.99	78	0.32

*Abbreviation*: *BMI* body mass index, *HDR* headache diary result, *VAS* visual analog scale

All values are presented as mean (SD)

^a^Received zinc capsule containing 220 mg zinc sulfate one time per day

^b^Received placebo capsule containing lactose one time per day

^c^Significant reduction throughout the trial: obtained from paired-sample t-test

^d^Measured by VAS that ranked headache severity from 1 to 10

^e^Determined by formula: frequency × duration

^*^Obtained from independent sample t-test for the comparison of changes

**Table 4:** Adjusted changes in serum zinc concentrations, BMI, and characteristics of migraine attacks in migraine patients who received either zinc supplement or placebo

	Zinc group^a^	Placebo group^b^	F	df	*P*-value^*^
BMI (kg/m^2^)	−0.22 ± 0.15	−0.05 ± 0.14	0.63	1	0.43
Zinc (μg/dL)	3.58 ± 1.19	3.15 ± 1.18	0.06	1	0.80
Headache severity^c^	−1.45 ± 0.30	−0.92 ± 0.29	1.35	1	0.24
Attacks frequency (per m)	−2.59 ± 0.71	0.09 ± 0.70	6.67	1	0.01
Attacks duration (hour)	−4.31 ± 1.53	−2.38 ± 1.51	0.75	1	0.38
HDR^d^	−56.44 ± 19.00	−14.62 ± 18.70	2.26	1	0.13

*Abbreviation*: *BMI* body mass index, *HDR* headache diary result, *VAS* visual analog scale

All values are presented as mean (SE), which were adjusted for baseline values, age, gender, BMI, and dietary intake of zinc throughout the trial

^a^Received zinc capsule containing 220 mg zinc sulfate one time per day

^b^Received placebo capsule containing lactose one time per day

^c^Measured by VAS that ranked headache severity from 1 to 10

^d^Determined by formula: frequency × duration

^*^Obtained from analysis of covariance (ANCOVA)

Participants reported no adverse effect following zinc and placebo supplementation. However, zinc supplementation improved appetite in some participants. Nevertheless, no significant effect of zinc supplementation on BMI was seen.

## Discussion

Currently, several therapeutic strategies such as relaxation training, butterbur, acupuncture, and B-vitamins supplementation have been suggested for the management of migraine symptoms [8–11]. In addition, dietary factors may have a role in migraine symptoms [28–30]. Zinc may affect these symptoms through its effects on neuronal function. However, to the best of our knowledge, no study has examined the effect of zinc supplementation on characteristics of migraine attacks.

Overall, we found that zinc supplementation resulted in a significant reduction in headache severity and migraine attacks frequency. However, this effect on headache severity became non-significant when baseline values of headache severity and potential confounders were taken into account. In line with our findings, Prasad et al. reported that zinc administration resulted in an improvement in the cold-related headaches [31]. Such finding was also reported in other two clinical trials [32, 33]. Furthermore, Evcili et al. reported that adherence to a low-glycemic index diet, characterized by a high amount of zinc, resulted in a significant reduction in the frequency of migraine attacks [34]. In a case-control study, migraine patients had a lower diet quality (defined by Healthy Eating Index) than those without migraine. Diet with a high quality contains a high amount of zinc [35].

It seems that zinc may increase the threshold of migraine attacks and reduce the frequency of migraine attacks through its effects on the nervous system [14–16]. Zinc affects the concentrations of neurotransmitters in the synaptic cleft probably through the action against neurotransmitter receptors and ion channels [36]. This element may inhibit the release of glutamate in the hippocampus [36]. Glutamate stimulates the N-methyl-D-aspartate (NMDA) receptor that is a key receptor in the initiation of migraine attacks [37, 38]. In addition, zinc may be a competitive antagonist for the NMDA receptor [39]. Moreover, zinc may inhibit the NMDA receptor via an increase in the concentrations of gamma-aminobutyric acid (GABA) that provides an NMDA-inhibitory effect [40, 41].

In the current study, we found no significant effect of zinc supplementation on headache severity, migraine attacks duration, and HDR. Unlike our findings, some clinical trials revealed that zinc supplementation significantly reduced the duration and severity of cold-related headaches [31, 32]. In addition, adherence to a zinc-rich diet for 3 months resulted in a significant reduction in the severity of migraine attacks [34]. Differences between our findings and those obtained from the previous studies can be attributed to different types of headaches, different dosages of zinc used for interventions, and different lengths of interventions. In the current study, dietary intake of zinc throughout the trial was higher among the placebo group than the zinc group. Although we adjusted for this difference in the final analysis, the confounding effect of this difference may not be fully excluded. Higher dietary intake of zinc in the placebo group compared with the zinc group may attenuate our estimates on the effect of zinc supplementation on headache severity, attacks duration, and HDR. Furthermore, the duration of migraine attacks and HDR at the study baseline were higher in the placebo group compared with the zinc group. This difference may be another reason for the observed non-significant effect of zinc supplementation on the duration of migraine attacks and HDR. Hence, further well-design clinical trials are needed to reveal the true effect of zinc supplementation on the mentioned symptoms.

As the strengths of the current study, we controlled for baseline values of outcome variables as well as some potential confounders such as BMI, age, gender, and dietary intake of zinc during the trial in our analysis. In addition, the compliance rate in our study was high, such that 100% of administered zinc and placebo capsules were taken throughout the trial. However, some limitations of our study should be taken into account. As a result of limited funding, we could not investigate the effect of zinc supplementation on inflammatory biomarkers that have a critical role in migraine symptoms [42]. In addition, we could not determine the effect of zinc supplementation on other migraine symptoms such as nausea, vomiting, and sensitivity to light and sound. Also, due to the small sample size of our study, we were unable to examine whether the favorable effect of zinc supplementation on the frequency of migraine attacks was different between men and women. Furthermore, the appropriate dosage of zinc affecting the frequency of migraine attacks cannot be inferred from this study, and further studies are required for this issue. Although we controlled the analysis for baseline values and some other confounders, further adjustments for residual confounders such as disease duration or the duration of the treatment without improvement were not possible due to the lack of data. Besides, we assessed the dietary intake of participants during the trial and controlled the analysis for dietary intake of zinc that was different between zinc and control groups; however, we could not entirely exclude the bias induced by the differences in dietary intakes of participants throughout the trial.

## Conclusion

We found a favorable effect of zinc supplementation on the frequency of migraine attacks among migraine patients. However, other characteristics of migraine attacks such as headache severity, migraine attacks duration, and HDR were not affected following zinc supplementation. Further clinical trials recruiting a homogenous group of migraine patients with a long duration of intervention are required in this area.

## Data Availability

The datasets analyzed during the current study are available from the corresponding author on reasonable request.
